# Supplementary figures and images for: Deciphering the genetic interactions between Pou4f3, Gfi1, and Rbm24 in maintaining mouse cochlear hair cell survival
Source: eLife. 2024 Mar 14;12:RP90025. doi: 10.7554/eLife.90025 (PMC10939501; doi:10.7554/eLife.90025)

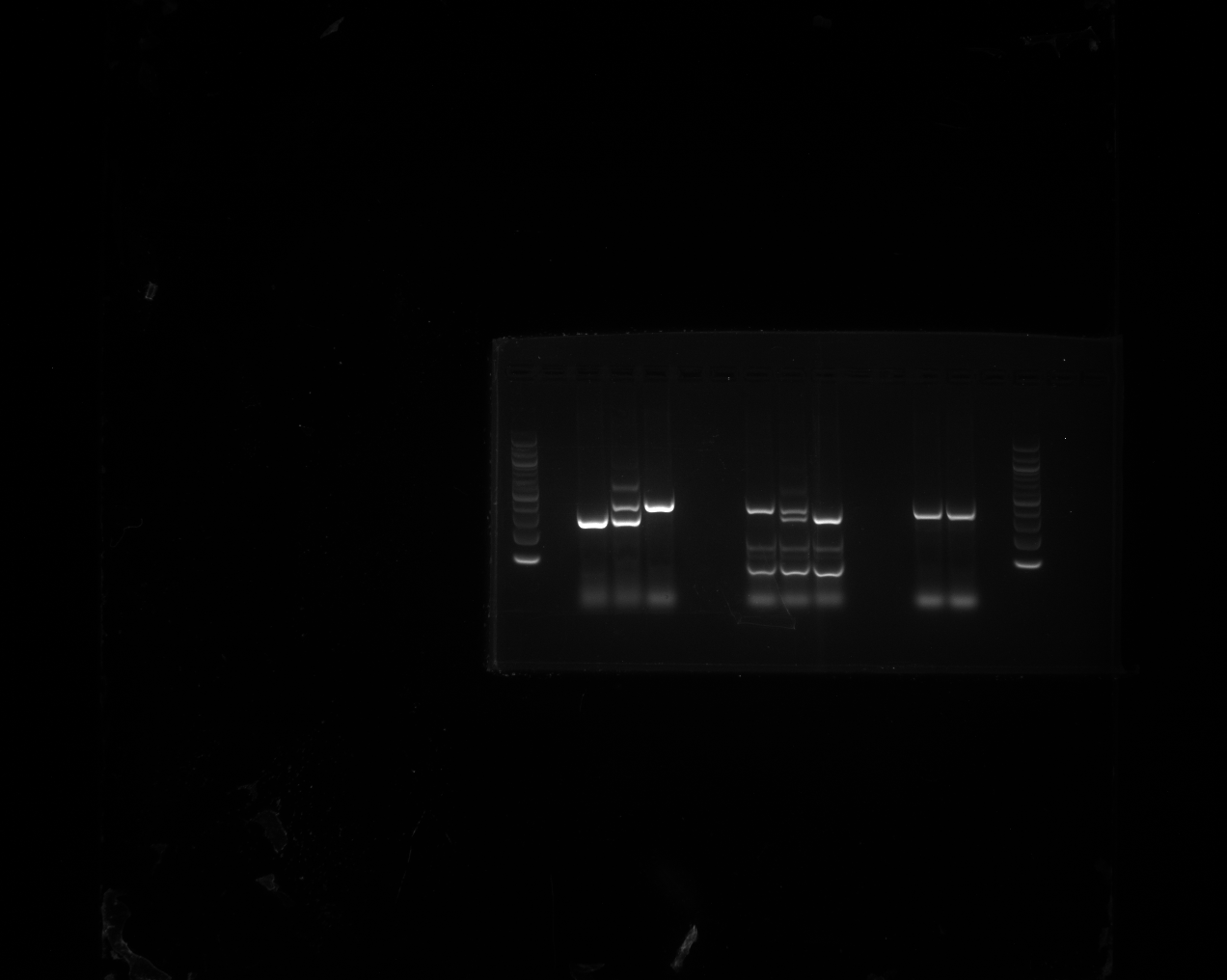

Supplement: Figure 1—source data 1. [file elife-90025-fig1-data1.zip › Figure 1-source data 1/Figure 1-source data 1.tif]

Figure 1

B

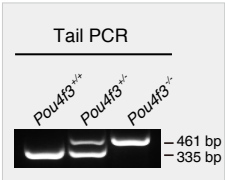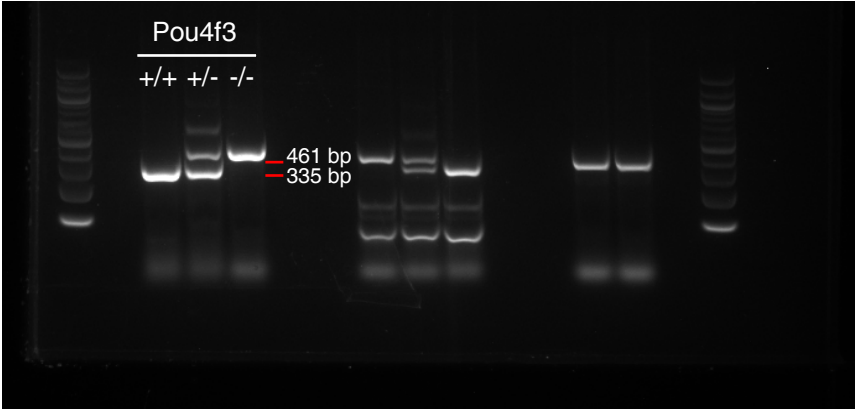

Supplement: Figure 1—source data 2. [file elife-90025-fig1-data2.zip › Figure 1-source data 2/Figure 1-source data 2.pdf]

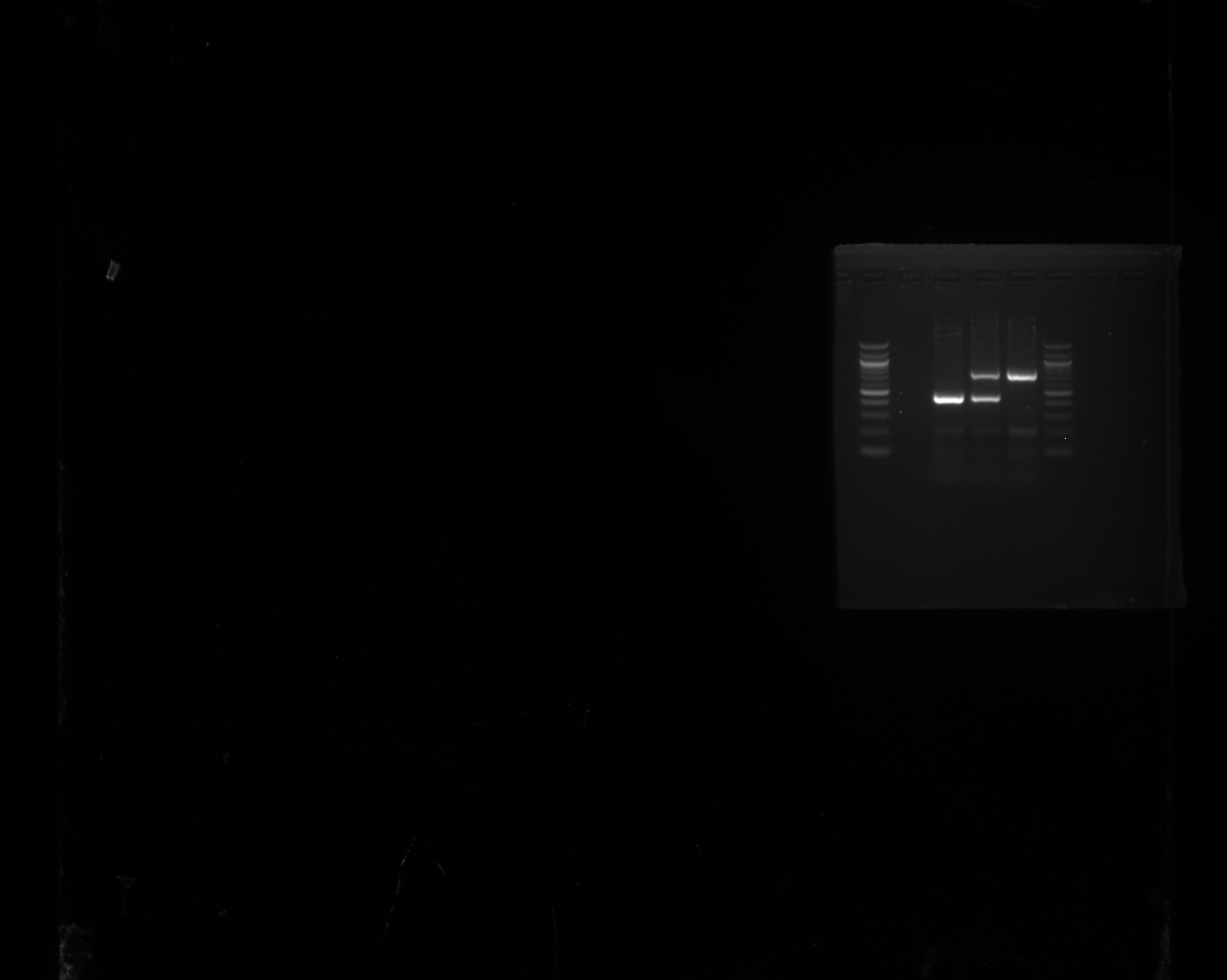

Supplement: Figure 2—figure supplement 1—source data 1. [file elife-90025-fig2-figsupp1-data1.zip › Figure 2-figure supplement 1-source data 1/Figure 2-figure supplement 1-source data 1.tif]

D

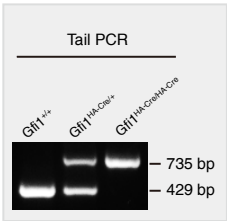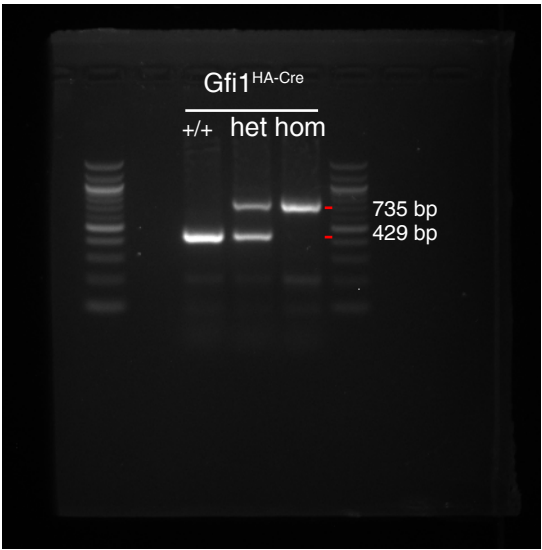

Supplement: Figure 2—figure supplement 1—source data 2. [file elife-90025-fig2-figsupp1-data2.zip › Figure 2-figure supplement 1-source data 2/Figure 2-figure supplement 1-source data 2.pdf]

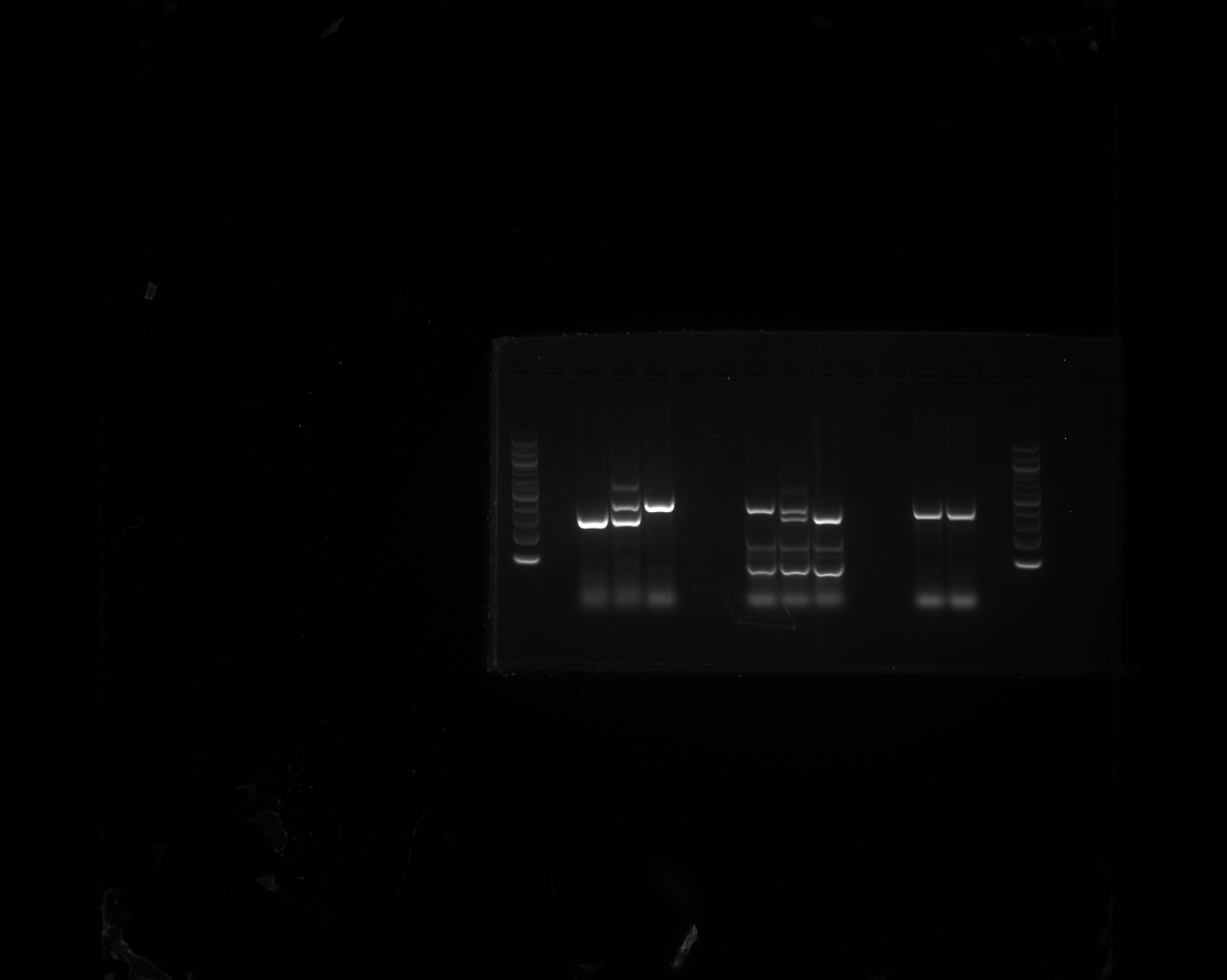

Supplement: Figure 2—figure supplement 3—source data 1. [file elife-90025-fig2-figsupp3-data1.zip › Figure 2-figure supplement 3-source data 1/Figure 2-figure supplement 3-source data 1.tif]

B

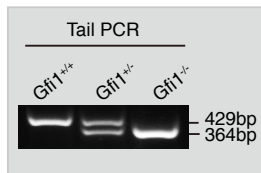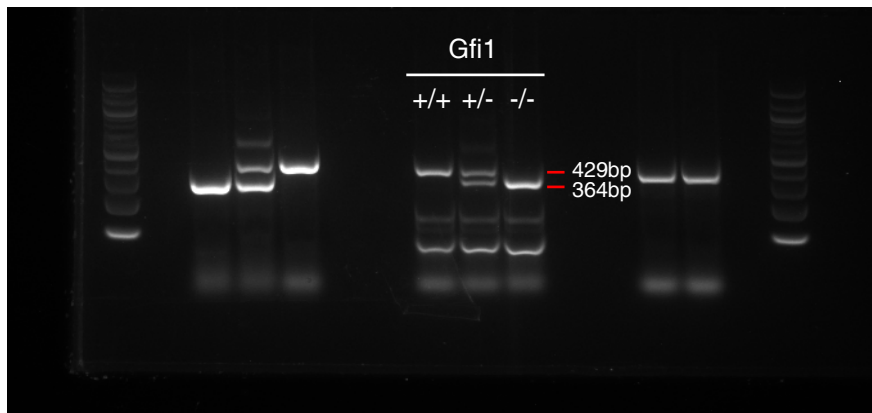

Supplement: Figure 2—figure supplement 3—source data 2. [file elife-90025-fig2-figsupp3-data2.zip › Figure 2-figure supplement 3-source data 2/Figure 2-figure supplement 3-source data 2.pdf]

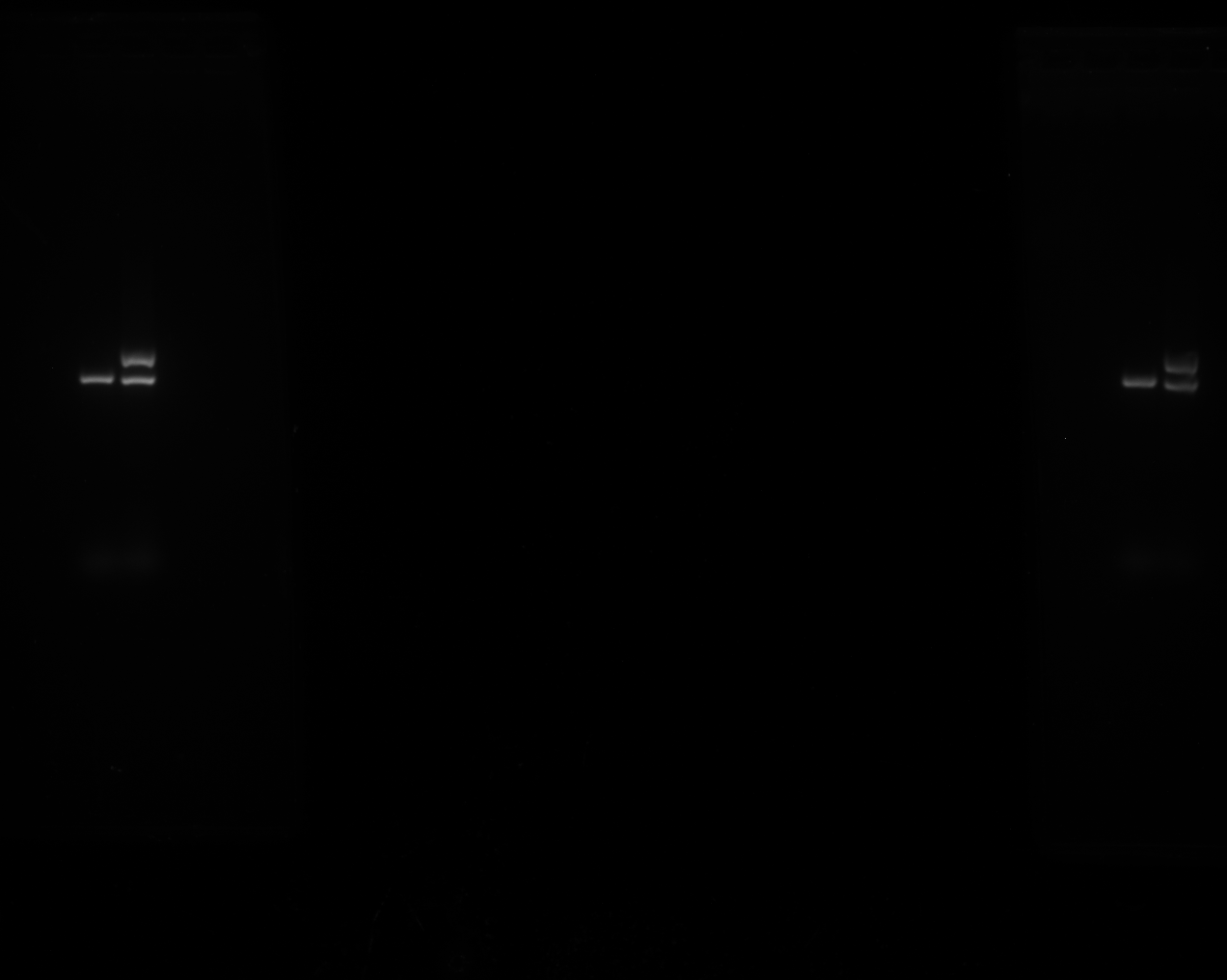

Supplement: Figure 5—source data 1. [file elife-90025-fig5-data1.zip › Figure 5-source data 1/Figure 5-source data 1.tif]

Figure 5

B

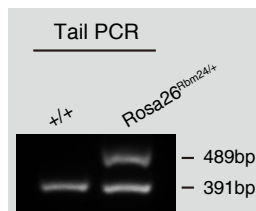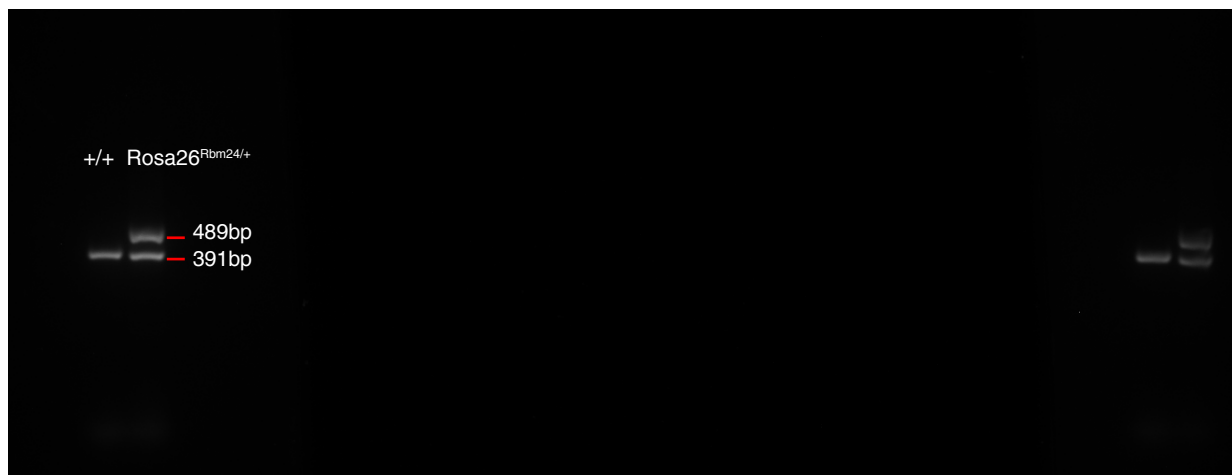

Supplement: Figure 5—source data 2. [file elife-90025-fig5-data2.zip › Figure 5-source data 2/Figure 5-source data 2.pdf]
